# Ultranarrow-bandwidth filter based on a thermal EIT medium

**DOI:** 10.1038/s41598-018-26215-9

**Published:** 2018-05-21

**Authors:** Gang Wang, Yu-Sheng Wang, Emily Kay Huang, Weilun Hung, Kai-Lin Chao, Ping-Yeh Wu, Yi-Hsin Chen, Ite A. Yu

**Affiliations:** 1 Department of Physics, National Tsing Hua University, Hsinchu, 30013 Taiwan; 2 College of Physics, Jilin University, Changchun, 130012 China; 3 Department of Physics, National Sun Yat-Sen University, Kaohsiung, 80424 Taiwan

## Abstract

We present high-contrast electromagnetically-induced-transparency (EIT) spectra in a heated vapor cell of single isotope ^87^Rb atoms. The EIT spectrum has both high resonant transmission up to 67% and narrow linewidth of 1.1 MHz. We get rid of the possible amplification resulted from the effects of amplification without population inversion and four-wave mixing. Therefore, this high transmitted light is not artificial. The theoretical prediction of the probe transmission agrees well with the data and the experimental parameters can be derived reasonably from the model. Such narrow and high-contrast spectral profile can be employed as a high precision bandpass filter, which provides a significant advantage in terms of stability and tunability. The central frequency tuning range of the filter is larger than 100 MHz with out-of-band blocking ≥15 dB. This bandpass filter can effectively produce light fields with subnatural linewidth. Nonlinearity associating with the narrow-linewidth and high-contrast EIT profile can be very useful in the applications utilizing the EIT effect.

## Introduction

A high-contrast electromagnetically-induced-transparency (EIT) medium can be realized as an atomic high-precision bandpass filter^1–5^. This filter can effectively produce a narrow-bandwidth light field and precisely tune the light frequency by selecting the atomic transition. Therefore, it is very valuable in the preparation of photon sources for possible future applications in quantum information precessing^6,7^. In practical applications utilizing EIT-based nonlinear optical responses, recently, an increasing number of researches have turned to the study in warm atomic media^8–12^. Such systems reduce the complexity of experimental setup and increase the repetition rate of quantum devices. An earlier study obtained an ultranarrow EIT linewidth in an anti-relaxation-coated cell in order to reduce the ground-state decoherence rate^13^. Very recently, near-unity EIT transmission efficiency has been reported in the microwave regime^14^. However, an EIT spectrum simultaneously satisfying the condition of narrow linewidth, high peak transmission, and low baseline transmission has not been presented to the best of our knowledge. Here, we report a high-contrast spectrum with subnatural linewidth of 1.1 MHz, EIT peak transmission of up to 67%, and the baseline transmission as low as 3%. Such excellent EIT characteristics advance the realization of optical memory using slow light.

An EIT spectrum can predict the features of optical memory or photon sources. The probe field propagating in the EIT medium induces a dramatically spatial compression, accompanied with ultraslow group velocity. The light field is possible to be stored in the medium as an optical memory^15^. Storage of light in an atomic vapor has been experimentally accomplished in 2001 with storage time of up to 0.5 ms in a magnetically-shielded and buffer-gas-filled cell^9^. This work was then extended into the quantum regime of single photons^16,17^. The efficiency for the storage and retrieval of light pulses can be optimized by applying time-reversal procedure in a low optical density system^18,19^. In addition, for the research of biphoton generation in an EIT-based spontaneous four-wave-mixing (SFWM) process^20–23^, the EIT linewidth and peak transmission correspond to the bandwidth and generation rate of the single-photon sources, respectively. The generated photon is engineered to match the wavelength and the bandwidth of the atomic transition, and it would be a perfect information carrier which well fits the requirements for the EIT-based optical memory^24^. These researches pave the avenue for the realization of quantum devices in Doppler-broadening media.

The characterizations of a high-performance EIT-based memory are the long delay time and high transmission of slow-light pulse. The optical density of the system (OD, denoted as *α*) is determined by the resonant probe field transmission. We can estimate the optical delay time from the EIT linewidth (*W*) and optical density, which is proportional to $\sqrt{\alpha }/W$ under the perturbation limit for Doppler-free media^25^. The EIT resonant transmission is another factor to determine the optical memory efficiency. We will provide a theoretical model by considering the velocity distribution of Maxwell-Boltzmann function and optical pumping effect. The simulation well fits the measured spectra and the experimental parameters such as effective OD and laser field Rabi frequency can be reasonably derived from the fitting. Hence, the spectral measurements and theoretical model advance our knowledge in the thermal-EIT study.

## Results and Discussion

### EIT spectra

**Figure 1:**
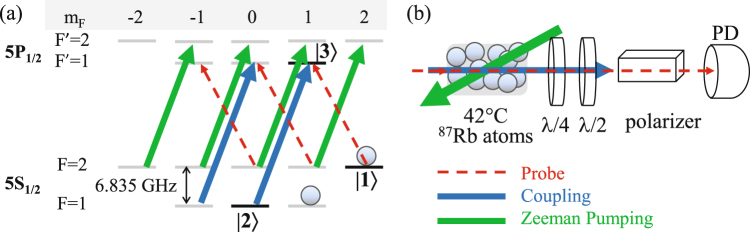
(**a**) Energy level diagram for ^87^Rb. The Zeeman pumping and the coupling fields pump the population to two Zeeman dark states. The *σ*^−^-polarized probe field and the *σ*^+^-polarized coupling field form a Λ-type EIT. (**b**) Schematic of the experimental setup. The Zeeman pumping beam propagates in an opposite direction with respect to the EIT pair. The Zeeman pumping and coupling fields have beam sizes (full width at *e*^−2^ maximum) of 4.0 mm and 2.8 mm (or 2.0 mm), respectively, making them uniform in the interaction regime within the probe field which has the beam size of only 0.71 mm. The vapor is heated up to 42 °C to increase the atomic density. In order to detect the weak probe field signal, two waveplates and one polarizer are arranged after the thermal cell to filter out the strong coupling field. *λ*/4 and *λ*/2: quarter and half waveplates, respectively; PD: photo detector.

**Figure 2:**
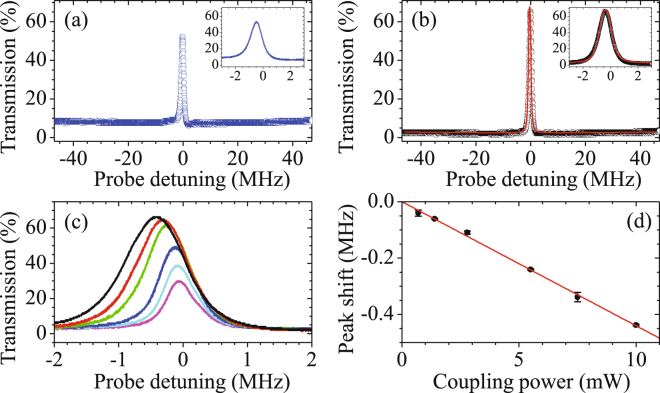
Typical EIT spectra in the absence of Zeeman pumping field in (**a**) and in the presence of that field in (**b**). The EIT spectrum in (**b**) shows a subnatural linewidth of around 1.1 MHz and a peak transmission of 67%. Red solid curve in (**b**) is the theoretical prediction from Eq. (3) under a set of *α* = 225 and (Γ_OP_, Ω_*c*_, *γ*) = (90, 4.2, 0.022)Γ. The peak transmission is getting higher with increasing coupling power, shown in (**c**). The applied powers of the coupling field are 10, 7.5, 5.5, 2.8, 1.4, and 0.70 mW from high to low peak transmissions. The corresponding frequency shifts of EIT peaks are presented in (**d**). Red line is a linear fit as the guide for the eyes.

We perform a continuous-wave EIT spectrum study in a ^87^Rb-filled cell. With the help of a coupling field and a Zeeman pumping field, all populations accumulate at two Zeeman ground states |1〉 and |*F* = 1, *m*_*F*_ = 1〉, as shown in Fig. 1. The probe field and the coupling field drive the transitions from the ground states |1〉 and |2〉 to the same excited state |3〉, respectively, forming a Λ configuration of EIT. In each EIT spectrum measurement, we frequency lock the coupling laser and sweep the probe laser frequency via electro-optic modulation. Further details of data analysis can be found in Methods. In the absence of the Zeeman pumping beam, the populations are distributed among different degenerate Zeeman states of hyperfine level *F* = 2 and state |*F* = 1, *m*_*F*_ = 1〉, decreasing the optical density. Therefore, the spectrum has a higher floor level as shown in Fig. 2(a). In addition, the population in the state |*F* = 2, *m*_*F*_ = 0〉 does not participate in the EIT transition because of the absence of the corresponding coupling field transition. Instead, the atoms contribute to the absorption of the probe field at the resonant frequency, resulting in a lower EIT transmission.

With a sufficiently strong Zeeman pumping field, the population accumulates at two Zeeman dark states. Both the optical density and EIT peak height (defined as the difference between the transmissions of EIT peak and baseline) increase and become saturated when the power of the Zeeman pumping field is stronger than 5 mW. In the whole measurements, a power of 14 mW is applied. We obtain an EIT peak transmission as high as 67% and a full width at half maximum of EIT linewidth of 1.1 MHz, shown in Fig. 2(b). It is worth noting that the low baseline transmission of only 3% lasted over 100 MHz, implying a large optical density. Such excellent EIT spectral profile can be employed as a pre-filter of single-photon sources from other systems. The central frequency tuning range of the filter is larger than 100 MHz by varying the coupling field transition frequency and the out-of-band extinction is larger than 15 dB. In addition, the EIT peak frequency linearly shifts with varied coupling field power, as shown in Fig. 2(c,d). This can be explained by the AC Stark effect (see Methods). As the result of the high-precision spectrum, a tiny frequency shift of 30 kHz is resolvable. Therefore, we demonstrate an optical filter with ultranarrow bandwidth, flexible central frequency, high peak transmission, and high out-of-band extinction.

### The model

In order to gain more insight into the EIT spectrum, we provide a theoretical analysis by solving the optical Bloch equations (OBEs) and Maxwell-Schrödinger equation (MSE) under the perturbation limit^25^ (i.e. the Rabi frequency of the probe field Ω_*p*_ is much weaker than that of the coupling field Ω_*c*_). The outgoing probe field is derived from the thermally-averaged atomic coherence of ${\rho }_{31}^{(v)}$. For a group with velocity *v* = *v*$\hat{z}$ relative to the laboratory frame, the dynamics of atomic coherences in the atomic frame are written as the follows.

1a $${\partial }_{t}{\rho }_{21}^{(v)}=\frac{i}{2}{{\rm{\Omega }}}_{c}^{\ast }{\rho }_{31}^{(v)}+i({{\rm{\Delta }}^{\prime} }_{p}-{{\rm{\Delta }}^{\prime} }_{c}+i\gamma ){\rho }_{21}^{(v)},$$

1b $${\partial }_{t}{\rho }_{31}^{(v)}=\frac{i}{2}{{\rm{\Omega }}}_{c}{\rho }_{21}^{(v)}+\frac{i}{2}{{\rm{\Omega }}}_{p}-(\frac{{\rm{\Gamma }}}{2}-i{{\rm{\Delta }}^{\prime} }_{p}){\rho }_{31}^{(v)},$$

1c $$(\frac{1}{c}{\partial }_{t}+{\partial }_{z}){{\rm{\Omega }}}_{p}=i\frac{\alpha {\rm{\Gamma }}}{2L}\frac{\int f(v){\rho }_{31}^{(v)}g(v)dv}{\int f(v)dv}\mathrm{.}$$

Here *ρ*_*ij*_ is an element of the density-matrix operator in an EIT system, *γ* denotes the ground state decoherence rate, Γ is the spontaneous decay rate of the excited state which is 2*π* × 5.75 MHz for Rb *D*_1_-line transition, *c* is the speed of light in vacuum, and *L* is the length of the medium. The physical definition of *α* here represents the optical density resulting from the entire ensemble having the resonant absorption cross section, and it can be directly derived from the system condition *nσL*, where *n* is the atomic density and *σ* is the absorption cross section. For a given temperature *T*_*c*_, atomic velocities are described by the Maxwell-Boltzmann distribution, $f(v)=\exp [-\frac{m{v}^{2}}{2{k}_{B}{T}_{c}}]$, where *m* is the atomic mass and *k*_*B*_ is the Boltzmann constant. The atomic motion induces Doppler shifts, Δ_*p*_′ = *ω*_31_ *−* *ω*_*p*_ − *k*_*p*_*v* ≡ Δ_*p*_ − *k*_*p*_*v* and Δ_*c*_′ = *ω*_32_ −* ω*_*c*_ − *k*_*c*_*v* ≡ Δ_*c*_ − *k*_*c*_*v*, where *ω*_*ij*_ denotes the transition frequency between the energy levels |*i*〉 and |*j*〉 and _*ωp*(*c*)_ is the probe (coupling) laser frequency. Because *k*_*p*_ ≅ *k*_*c*_, Δ*p*′ − Δ*c*′ is replaced by Δ_*p*_ − Δ_*c*_ ≡* δ*, defined as two-photon detuning. In our measurements, Δ_*c*_ = 0 because of frequency locking.

Additionally, we consider the optical pumping effect that causes the atomic accumulation for different velocity groups. Strong Zeeman pumping and coupling fields pump more high-velocity groups to the ground state |1〉 with detunings of ${{\rm{\Delta }}^{\prime} }_{c}={k}_{c}v\gg {\rm{\Gamma }}$ (in the atomic frame). These high velocity groups then participate the EIT peak transition (around Δ_*p*_ = 0 and *δ* = 0) with large one-photon detunings Δ_*p*′_ = *k*_*p*_*v*; meanwhile at out-of-band detunings of ${{\rm{\Delta }}}_{p}={k}_{p}v\gg {\rm{\Gamma }}$ these velocity groups absorb the resonant probe field (because of Δ_*p*′_ = 0), resulting in a broader absorption spectrum. The selection of velocity groups is expressed as the power broadening function, $g(v)=\frac{1}{1+\mathrm{4(}{k}_{c}v{)}^{2}\,/\,{{\rm{\Gamma }}}_{{\rm{OP}}}^{2}}$. The optical pumping linewidth Γ_OP_ is nearly a linearly increasing function of the powers of the Zeeman pumping and coupling fields. In all of the measurements, the values of Γ_OP_ varied from 18Γ to 120Γ according to different laser intensities.

Based on the steady-state solution of the optical-Bloch equations, we derive ${\rho }_{31}^{(v)}$ from Eq. (1a) and (1b),

2 $$\frac{{\rho }_{31}^{(v)}}{{{\rm{\Omega }}}_{p}}=\frac{2i(\gamma -i{{\rm{\Delta }}}_{p})}{|{{\rm{\Omega }}}_{c}{|}^{2}+2(\gamma -i{{\rm{\Delta }}}_{p})[{\rm{\Gamma }}-2i({{\rm{\Delta }}}_{p}-{k}_{p}v)]}\equiv \sigma (v,{{\rm{\Delta }}}_{p}\mathrm{).}$$

The probe transmission is a function of the probe field detuning by inserting ${\rho }_{31}^{(v)}$ into Eq. (1c),

3 $$T({{\rm{\Delta }}}_{p})=\exp (\frac{-\alpha {\rm{\Gamma }}}{\sqrt{\pi }{{\rm{\Gamma }}}_{{\rm{D}}}}\int f(v\mathrm{)\ }{\rm{Im}}[\sigma (v,{{\rm{\Delta }}}_{p}\mathrm{)]\ }g(v\mathrm{)\ }{k}_{p}dv),$$

where ${{\rm{\Gamma }}}_{{\rm{D}}}=\sqrt{2{k}_{B}{T}_{c}{k}_{p}^{2}/m}=54{\rm{\Gamma }}$ for the cell temperature of 42 °C.

To fit the spectrum by the model, we first determine the optical density *α* and optical pumping linewidth Γ_OP_ from the baseline curve of the spectrum in a frequency range over ±50 MHz. The optical density *α* dominates the absorption depth and Γ_OP_ individually governs the curvature of absorption line. From the systematic study, a stronger Ω_*c*_ corresponds to a larger Γ_OP_ while the optical density remains nearly a constant. We consider 4001 velocity classes ranging from −200Γ to 200Γ. With the given *α* and Γ_OP_, Ω_*c*_ and ground state decoherence rate *γ* are resolved from the fitting of the EIT linewidth and peak height, respectively. The best fit of the measurement in Fig. 2(b) gives a set of *α* = 225 and (Γ_OP_, Ω_*c*_, *γ*) = (90, 4.2, 0.022)Γ. Compared with the estimation according to the system condition *nσL*, the determined *α* from the theoretical model is acceptable. As the probe field detuning is a little outside the transparency window, the baseline transmission is derived as *T*_baseline_ = exp$[-\alpha ({\rm{\Gamma }}/{{\rm{\Gamma }}}_{{\rm{D}}})(\sqrt{\pi }\mathrm{/2)]}$ by setting Ω_*c*_ and *γ* to zero and assuming ${\rm{\Gamma }}\ll {{\rm{\Gamma }}}_{{\rm{D}}}$ and ${\rm{\Gamma }}\ll {{\rm{\Gamma }}}_{{\rm{OP}}}$. The expression gives an effective optical density ${\rm{OD}}=\alpha ({\rm{\Gamma }}/{{\rm{\Gamma }}}_{{\rm{D}}})(\sqrt{\pi }\mathrm{/2)}=3.7$, which means only a small fraction of hot atoms with a small range of the velocity participate in the one-photon transition. Moreover, by considering the laser power, beam size, and atomic transition rate, we derive Ω_*c*_ = 4.25Γ, which is consistent with the theoretically determined one. Hence, the theoretical prediction fits the data well and the experimental parameters can be derived reasonably from the model.

We further simplify Eq. (3) under the assumptions of $\gamma \ll {\rm{\Gamma }}$ and $\gamma {\rm{\Gamma }}\ll {{\rm{\Omega }}}_{c}^{2}$ for an extremely small decoherence rate and ${{\rm{\Delta }}}_{p}{\rm{\Gamma }}\ll {{\rm{\Omega }}}_{c}^{2}$, ${{\rm{\Delta }}}_{p}{{\rm{\Gamma }}}_{{\rm{D}}}\ll {{\rm{\Omega }}}_{c}^{2}$ for a small frequency range around the EIT peak. The probe transmission is then modeled by

4 $$T({{\rm{\Delta }}}_{p})=\exp (\frac{-\alpha {\rm{\Gamma }}}{\sqrt{\pi }{{\rm{\Gamma }}}_{{\rm{D}}}}(\frac{2\gamma }{{{\rm{\Omega }}}_{c}^{2}}+\frac{4{{\rm{\Delta }}}_{p}^{2}{\rm{\Gamma }}}{{{\rm{\Omega }}}_{c}^{4}})\int {e}^{\frac{-{x}^{2}}{{{\rm{\Gamma }}}_{{\rm{D}}}^{2}}}\frac{1}{1+\frac{4{x}^{2}}{{{\rm{\Gamma }}}_{{\rm{OP}}}^{2}}}\frac{1}{1+\frac{4{x}^{2}}{{{\rm{\Gamma }}}_{{\rm{EIT}}}^{2}}}\,dx),$$

where Γ_EIT_ is defined as ${{\rm{\Omega }}}_{c}^{2}\mathrm{/2}\gamma$ and *k*_*p*_*v* is replaced by *x* in unit of Γ. The EIT peak transmission can be written as

5 $$T\mathrm{(0)}=\exp (-\frac{2\alpha \gamma {\rm{\Gamma }}}{{{\rm{\Omega }}}_{c}^{2}}{C}_{\alpha }({{\rm{\Gamma }}}_{{\rm{OP}}},{{\rm{\Gamma }}}_{{\rm{EIT}}})),\,{\rm{where}}\,{C}_{\alpha }({{\rm{\Gamma }}}_{{\rm{OP}}},{{\rm{\Gamma }}}_{{\rm{EIT}}})=\frac{1}{\sqrt{\pi }{{\rm{\Gamma }}}_{{\rm{D}}}}\int {e}^{-\frac{{x}^{2}}{{{\rm{\Gamma }}}_{{\rm{D}}}^{2}}}\frac{1}{1+\frac{4{x}^{2}}{{{\rm{\Gamma }}}_{{\rm{OP}}}^{2}}}\frac{1}{1+\frac{4{x}^{2}}{{{\rm{\Gamma }}}_{{\rm{EIT}}}^{2}}}{\rm{dx}}.$$

**Figure 3:**
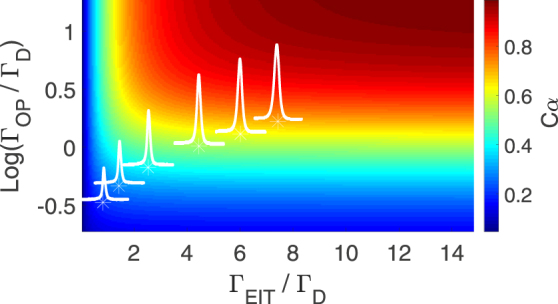
The calibration factor *C*_*α*_ for a Doppler-broadened medium is a function of optical pumping linewidth Γ_OP_ and EIT linewidth Γ_EIT_. The EIT peak transmission $T(0)=\exp [-\mathrm{(2}\alpha \gamma {\rm{\Gamma }}/{{\rm{\Omega }}}_{c}^{2}){C}_{\alpha }]$ with the effective OD of *αC*_*α*_. The six EIT spectra in Fig. 2(c) are also shown here with the corresponding Γ_OP_/Γ_D_ and Γ_EIT_/Γ_D_.

As mentioned before, the peak transition (Δ_*p*_ = 0) is contributed by all of velocity groups which mediate one-photon-detuning EIT transition. The physical definition of Γ_EIT_ here represents the linewidth in a velocity spectrum or one-photon-detuning spectrum. The EIT peak *T*(0) in a Doppler-broadened medium has the similar expression $\exp [-2\alpha \gamma {\rm{\Gamma }}/{{\rm{\Omega }}}_{c}^{2}]$ (which has been widely used in a Doppler-free ensemble) with a calibration factor *C*_*α*_. The finite EIT linewidth and optical pumping linewidth degrade the effective optical density. The calibration factor *C*_*α*_ as a function of Γ_OP_ and Γ_EIT_ is plotted in Fig. 3. Take the parameters of Fig. 2 for example (Γ_OP_/Γ = 90 and Γ_EIT_/Γ = 400), we derive *C*_*α*_ = 0.69, which means the effective optical density of the EIT transmission *αC*_*α*_ = 157. We further discuss the propagation velocity of the probe pulse in this optically thick media. The phase of the probe field *ϕ* is expressed as

6 $${\phi }=\frac{-\alpha {\rm{\Gamma }}}{2\sqrt{\pi }{{\rm{\Gamma }}}_{{\rm{D}}}}\int f(v)\,{\rm{Re}}[\sigma (v,{{\rm{\Delta }}}_{p})]\,g(v)\,{k}_{p}dv\mathrm{.}$$

We derive the group delay time of the slow-light pulse as $\alpha {C}_{\alpha }{\rm{\Gamma }}/{{\rm{\Omega }}}_{c}^{2}$ from *dϕ*/*d*Δ_*p*_ at Δ_*p*_ = 0. The expression is similar to the one widely used in cold ensembles^25^. For a Doppler-free medium, the optical pumping linewidth and EIT linewidth are both sufficiently broader than Γ_D_. Thus, *C*_*α*_ reaches unity. All of the populations are prepared to the dark states and then participate the EIT transition. On the other hand, if the linewidths are both sufficiently narrow, the effective optical density is only contributed from the Doppler-free atoms. The values of the above-mentioned linewidths provide the useful information on the range of velocity groups which need to be taken into account on the EIT transition. This model advances the knowledge in the thermal-EIT study.

### Properties of EIT spectra

**Figure 4:**
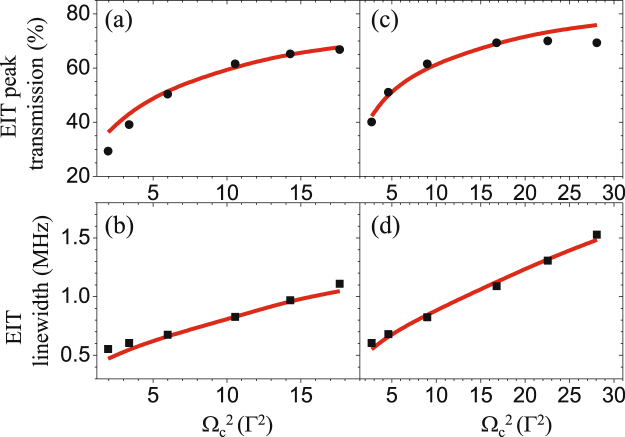
EIT peak transmission and linewidth with varied coupling field power. The beam size ratios of the coupling and probe fields are 4.0 (in (**a**) and (**b**)) and 2.8 (in (**c**) and (**d**)). The prediction curves, shown in red, are calculated by Eq. (3) under fixed parameters of *α* = 225 and *γ* = 0.022 Γ. Ω_*c*_ and Γ_OP_ are determined by the measured spectra and both are nearly linear functions of the coupling power.

The expression of EIT peak transmission shows that a stronger coupling field leads to a higher transmission. We systematically study the EIT transmission with varied coupling field power. At each condition, the baseline transmission keeps at the same low level, indicating the optical density does not vary with Ω_*c*_. As Ω_*c*_ gets stronger, corresponding to larger Γ_OP_ and Γ_EIT_, the EIT peak height and linewidth become higher and broader. The EIT peak transmission (circles) and linewdith (squares) with varied Ω_*c*_ are shown in Fig. 4(a,b). We further increased Ω_*c*_ by reducing the coupling beam size to 2.0 mm. The results are shown in Fig. 4(c,d). The EIT peak saturates at 70%. Further increasing Ω_*c*_ does not enhance the peak transmission but it does broaden the EIT linewidth. For the applications in optical filters or in biphoton generations via SFWM process^20–23^, the bandwidth of the filter or the linewidth of photon source is controllable while the central frequency maintains a high transmission.

The theoretical predictions with fixed optical density *α* = 225 and decoherence rate *γ* = 0.022Γ well fit the data of EIT peak transmission and linewidth, as the shown red curves in Fig. 4. At strong Ω_*c*_ regime, the peak height goes lower than the predicted value. The discrepancy is caused by the impurity of the coupling field polarization due to the photon switching effect^26^. The *σ*^−^ coupling field destroys the quantum interference of EIT and thereby results in absorption of the probe field. In addition, the peak height at a weak Ω_*c*_ condition is also lower than the prediction because of the EIT transient effect. A weak Ω_*c*_ leads to a long EIT response time^27^. As the result of the finite atomic transient time that atoms move in and out of the interaction regime, EIT has not reached the steady-state condition, implying the degradation of the EIT peak height. Thus, the theoretical prediction supports the experimental observations and physical picture.

Moreover, for the purpose of photon source generation, we should get rid of any amplification. Once the beam size of the coupling field is too small, the population transient effect needs to be taken into account: The fresh atoms, entering the probe interaction region, is not optically pumped to the Zeeman dark states in advance, and therefore, the probe field is amplified through the so-called amplification without population inversion (AWI)^28^. A smaller coupling beam size can potentially make the AWI more prominent. At the same value of Ω_*c*_, the peak transmission of the coupling beam size of 2.8 mm shown in Fig. 4(a) is less than that of 2.0 mm shown in Fig. 4(b) by less than 2.5%. The amplification due to the AWI effect is very insignificant in the data of 67% peak transmission shown in Figs 2(b) and 4(a). Furthermore, the four-wave-mixing (FWM) amplification, in which the coupling field also excites the population in the ground state driven by the probe field^29^, is not allowed in our experiment. This is because the coupling and probe fields need to have the same polarization to induce this amplification, but they have the orthogonal-polarization configuration here. We further exclude the amplification induced by a little impurity of the *σ*^−^ polarization of Zeeman pumping field via another kind of FWM process^30,31^. When the atomic transitions involve any gain effect, the determined decoherence rate *γ* should go lower. To test whether there could be any gain effect, we adjust the polarization ratio of *σ*^−^ to *σ*^+^ components of the Zeeman pumping field, whose Rabi frequencies are denoted as ${{\rm{\Omega }}}_{{\rm{ZP}}}^{-}$ and ${{\rm{\Omega }}}_{{\rm{ZP}}}^{+}$, respectively. When the ratio ${({{\rm{\Omega }}}_{{\rm{ZP}}}^{-}/{{\rm{\Omega }}}_{{\rm{ZP}}}^{+})}^{2} < 0.03$, *γ* did not change; and once the ratio became 0.06, *γ* increased by 6%. Since ${({{\rm{\Omega }}}_{{\rm{ZP}}}^{-}/{{\rm{\Omega }}}_{{\rm{ZP}}}^{+})}^{2}$ in our system is less than 0.01, the FWM gain does not occur in our system. Hence, we believe the high EIT transmission is not artificial and such a high-contrast EIT spectrum would be useful in the future applications.

## Conclusion

We systematically investigate the thermal-EIT spectra which can make a quality prediction for a slow light or photon source. The spectral profile shows a high EIT peak transmission of 67%, a narrow EIT linewidth of 1.1 MHz, and a low off-resonant transmission less than 3%. We get rid of the possible amplification, and hence this high transmitted light is not artificial. A high-contrast EIT medium can be applied as an ultranarrow-bandwidth filter. The central frequency of the filter can be precisely tuned, making it flexible in the generation of photon sources with subnatural linewidth. We further provide a theoretical model to simulate EIT spectra. The prediction fits the data well and the experimental parameters can be reasonably derived from the model. Hence, the spectral measurements and theoretical model advance our knowledge in the thermal-EIT study.

## Methods

### Setup and Measurements

We perform a continuous-wave EIT spectrum study in a ^87^Rb-filled cell (Thorlabs GC25075-RB). All of the laser fields drive *D*_1_-line transition at wavelength of 795 nm. With the help of the coupling field (which couples the transition between states |*F* = 1〉 and |*F*′ = 1〉 and the Zeeman pumping field (which couples that between states |*F* = 2〉 and |*F*′ = 2〉), all populations accumulate at two Zeeman ground states |1〉 and |*F* = 1, *m*_*F*_ = 1〉, as shown in Fig. 1. The coupling field is produced by an external cavity diode laser (ECDL). One beam from the ECDL is sent through an electro-optic modulator (EOM) before injection locking the probe field. The probe and coupling beams are nearly collinear propagating to reduce two-photon Doppler broadening (*k*_*c*_ − *k*_*p*_Cos*θ*)*v*, where *k*_*c*(*p*)_ represents the wavevector of the coupling (probe) field, *θ* is the angle between them, and *v* is the atomic velocity. The Zeeman pumping beam has a sufficiently large intersecting angle of around 0.9° with respect to EIT beams to avoid any light leakage into the detector. We apply a serial waveplates and crystal polarizer to separate the probe field (*σ*^−^ polarization) from the coupling beam (*σ*^+^ polarization) after cell. This polarizer filter reduces the coupling field by 48 dB while the probe field keeps 85% transmittance. In order to observe single-photon-level EIT signals or biphton signals^20–23^, one can further apply multiple spectral filters (e.g. Fabry-Perot etalons) to diminish the stray light.

### Data Analysis

For each EIT spectrum, we normalize the probe transmission by the incident power. In the absence of the coupling field, 0.6% of the probe field can be no longer absorbed at a further larger optical density medium by heating up the cell temperature. This component came from the sideband signal of the probe laser, which was injection-locked by the coupling laser after an EOM. It has to be subtracted for the calculation of the probe field transmission. The incident power of the probe field was measured at a far-off-resonant frequency (6.8-GHz red-detuned respective with the resonant transition of |1〉 to |3〉). The applied power of the probe field was typically 2.4 *μ*W right before the cell and the maximum power of the coupling beam was 10 mW.

### AC Stark Shifts

As shown in Fig. 2(c,d), the central frequency of EIT peak linear shifts with the applied coupling field power due to the AC Stark effect. The coupling field also couples the far-off-resonant transition between states |*F* = 〉 and |*F*′ = 2〉 with detuning of 814.5 MHz. We suppose that the velocity group of *v* = 0 dominates the transition so that Δ_*AC*_ = 814.5 MHz is not a velocity-dependent function. We further assume that the coupling field only drove one far-off-resonant transition from state |2〉 to state |*F*′ = 2, *m*_*F*_′ = 1〉. The transition has a Clebsch-Gordan coefficient that is $\sqrt{3}$ times as lager as that of of |2〉 to |3〉 transition. The amount of AC Stark shift for the spectrum in Fig. 2(b) is only 20% different from the estimated value of ${(\sqrt{3}{{\rm{\Omega }}}_{c})}^{2}\mathrm{/4}{{\rm{\Delta }}}_{AC}$.
